# Gel-Based Nanocarrier for Intravesical Chemotherapy Delivery: In Vitro and In Vivo Study

**DOI:** 10.3390/ph13110329

**Published:** 2020-10-22

**Authors:** Ting-Yu Chen, Ming-Jun Tsai, I-Ling Lin, Li-Ching Chang, Pao-Chu Wu

**Affiliations:** 1 School of Pharmacy, College of Pharmacy, Kaohsiung Medical University, Kaohsiung 80708, Taiwan; sylviab074@gmail.com; 2 Department of Neurology, China Medical University Hospital, Taichung 406040, Taiwan; d22570@mail.cmuh.org.tw; 3 School of Medicine, Medical of College, China Medical University, Taichung 406040, Taiwan; 4 Department of Neurology, An-Nan Hospital, China Medical University, Tainan 70965, Taiwan; 5 Department of Medicine Laboratory Science and Biotechnology, College of Health Science, Kaohsiung Medical University, Kaohsiung 80708, Taiwan; linili@kmu.edu.tw; 6 School of Medicine for International Students, I-Shou University, Kaohsiung 82445, Taiwan; 7 Department of Medical Research, Kaohsiung Medical University Hospital, Kaohsiung 80708, Taiwan; 8 Drug development and value creation research center, Kaohsiung Medical University, Kaohsiung 80708, Taiwan

**Keywords:** intravesical administration, nanocarrier, chemotherapeutic agent, drug delivery carriers

## Abstract

Intravesical administration of chemotherapeutic agents can enhance drug accumulation in tumors and reduce systemic side effects. Nanocarriers were developed for intravesical administration and exploit the permeation enhancement effect. In vitro permeation evaluation, the drug transdermal amount and accumulation amounts in the tissue of gemcitabine-loaded nanocarriers through biological membrane significantly increased about 14.8~33.0-fold and 1.5~14.1-fold respectively, when compared to a control group of 1% gemcitabine saline solution. In in vivo intravesical administration, the drug accumulation amount in bladder tissue of nanocarrier of 75.2 ± 5.4 μg was revealed as being comparably higher than that of the control group of 44.8 ± 6.4 μg. In confocal laser scanning microscopy imagery, the penetration depth of fluorescent dyes-rhodamine was increased from 80 μm up to 120 μm when a nanocarrier was used. This result implies that the nanocarrier is a promising drug delivery agent for intravesical administration.

## 1. Introduction

Bladder cancer is a common genitourinary malignancy involving the urinary system, and is commonly divided into non-muscle invasive or superficial bladder cancer (approximately 70%) and muscle-invasive bladder cancer where the cancer extends into the underlying smooth muscle [1,2]. The treatment of superficial bladder cancer involves transurethral resection of visible tumors followed by chemotherapy for the prevention of recurrent tumors. The intravesical chemotherapy agent provides high drug concentrations at the disease site, and minimum systemic side effects due to the theoretically negligible systemic uptake. Unfortunately, intravesical instillation administration has limited efficacy due to obstacles of the urothelial epithelium, and regular replacement and flushing during urine formation and elimination. Hence, new and more effective therapeutic strategies have been investigated [3,4].

Nanotechnology is an emerging field, and provides new tools and techniques for drug delivery systems in the pharmaceutical industry. The most common nanoscale carriers such as nanoparticles, liposomes and microemulsions have been used to enhance drug therapeutic efficacy [4,5,6], including increasing the solubility and improving biological membrane permeability and uptake/permeation of therapeutic compounds into malignant tissues. Microemulsion is a useful nanocarrier in the pharmaceutical field, because its composition (an aqueous phase, oil phase, surfactant and often cosurfactant) is simple, and easy to prepare. Microemulsion incorporated hydrophilic polymer to form gel-based microemulsion, which could increase the retention time of formulations on the administration site, result in increased therapeutic efficacy. Hence, the gel-based nanocarriers were designed for intravesical administration. In general, the formation, physicochemical properties, and effectiveness of the designed formulation were significantly affected by the composition proportion of formulations. In this study, the response surface methodology (RSM) with a constrained mixture design was used to realize the effect of surfactant and cosurfactant of microemulsion and we obtained an optimal formulation.

Gemcitabine hydrochloride is a water-soluble deoxycytidine analog with widely antitumor activity. In the cell, it was phosphorylated, and incorporated into RNA and DNA, thereby inhibiting growth activity and mediating apoptosis. It has been shown to be effective and well-tolerated for superficial bladder cancer treatment [7,8]. Hence, gemcitabine was used as the model chemotherapy drug in this study, to evaluate the permeability enhancement effect of designed gel-based nanocarriers through in vitro permeation study and in vivo intravesical infusion test.

## 2. Results and Discussion

### 2.1. Characteristics of Formulations

Eight model gemcitabine-loaded nanocarrier formulations were prepared as per the mixture experimental design. The mean droplet size, polydispersity index and viscosity of all formulations were measured and are presented in Table 1. The polydispersity index of all formulations ranged from 0.05 to 0.35. Most of them were less than 0.2 except for formulation F03, indicating that the mono-dispersity of the nanocarrier was obtained [9]. The mean droplets were in the nanoscale range, ranging from 10.5 to 163.5 nm. Previous studies have pointed out that only particles with a size of 50 to 500 nm are more likely to penetrate physiological membranes [10]. The viscosities ranged from 274.1 to 483.0 cps.

### 2.2. In Vitro Permeation and Accumulation Study

**Figure 1:**
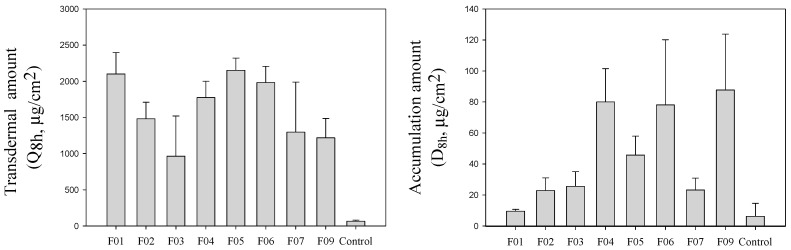
The permeability parameters of model gemcitabine-loaded formulations and control group (1% gemcitabine solution).

The drug transdermal amount in receptor cell (Q_8h_), and drug accumulation amount in the membrane (D_8h_) of gemcitabine-loaded microemulsion formulations after 8 h applied are depicted in Figure 1. A Control group of 1% gemcitabine solution was used to demonstrate the penetration enhancement effect of experimental formulations. The Q_8h_, and D_8h_ of the control group were 65.3 ± 15.3 μg/cm^2^ and 6.2 ± 8.4 μg/cm^2^ respectively. In the case of microemulsion formulations, the Q_8h_ and D_8h_ were remarkably enhanced about 14.8~33.0-fold and 1.53~14.1-fold, respectively. This finding agreed with earlier studies reporting that drug permeability can be remarkably enhanced by using nanoscale carriers [11,12,13,14,15,16,17,18].

To evaluate the influence degree of each independent variable on the respective combination as response term (Q_8h_, D_8h_), the mathematical relationships were generated using multiple linear regression analysis. The optimal model was chosen and validated through the determination of model factorial ANOVA analysis (*p*-value < 0.05) and the lack of fit significance (relative to the pure error, *p*-value > 0.05). The best models were as following:

Ln (Q_8h_) = 7.55 X_1_ + 7.71 X_2_ + 2.35 X_3_ (1)

Ln (D_8h_) = 4.55 X_1_ + 5.07 X_2_ + 71.12 X_3_ − 5.07 X_12_ − 88.95 X_13_ − 85.32 X_23_ (2)

**Figure 2:**
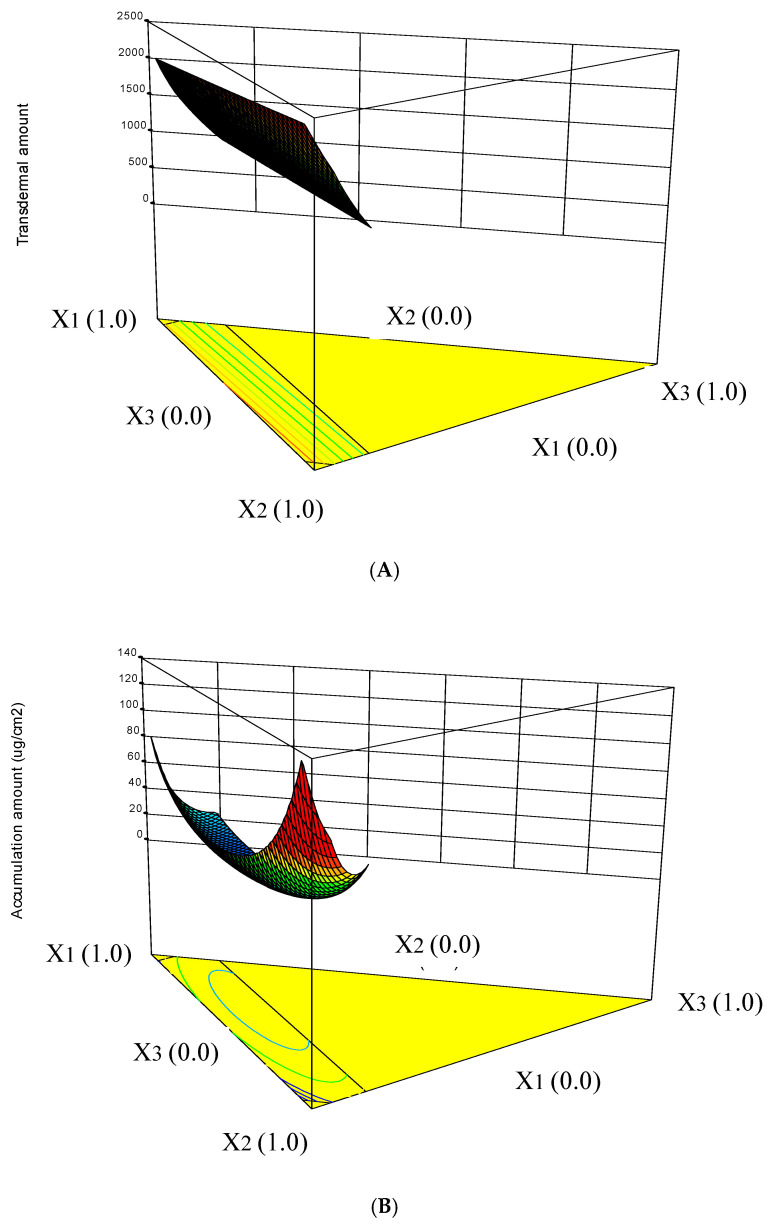
Three dimensional response surface diagrams for the influence of formulation variables on the transdermal amount (**A**) and accumulation amount in tissue (**B**).

The three-dimensional response surface diagrams describing the interaction effects of the independent variables on the responses are shown in Figure 2. It was found that X_2_:carbitol had the greatest influence on the drug transdermal cumulative amount. Carbitol is a very good solvent for many compounds that are poorly soluble in either water or oil. It is also a potential permeation enhancer that can improve the vesicular bilayer fluidity and reducing the biological membrane barrier [17] (Figure 2A). In the drug accumulation amount in tissue (Figure 2B), the interaction of X_13_ and the main factor of X_3_ (benzalkonium chloride) showed the greatest influence effect. Benzalkonium chloride, a quaternary ammonium antimicrobial agent, is also a potential penetration enhancer [18,19]. Finally, an appropriate formulation with higher Q_8h_ and D_8h_ was obtained by the optimization process and subject to in vivo intravesical infusion test.

### 2.3. In Vivo Intravesical Instillation of Gemcitabine

To evaluate the efficacy of the nanocarrier, the plasma concentration, drug accumulation in bladder tissue and the penetration depth were determined one hour later following intravesical administration. The gemcitabine accumulation amounts in the bladder were 44.8 ± 6.4 μg and 75.2 ± 5.4 μg for the control group and nanocarrier respectively. The tissue accumulation amount of nanocarrier formulation was significantly increased (*p* < 0.05), indicating that the nanocarrier exhibited potential penetration capacity. The results may be attributed to (1) the nanocarrier having increased thermodynamic activity of the therapeutic compounds thereby enhancing its partitioning into the biological membrane; and (2) the components of the formulation, particularly surfactant and cosurfactant acting as penetration enhancers to reduce the diffusional barrier of the membranous epithelium and enhance the drug permeability through epithelium membranous [20,21,22].

The plasma drug concentration was 6.74 ± 1.32 μg/mL after the control group application, indicating that slight amounts of gemcitabine were absorbed through the mucosa. The plasma concentration of 7.33 ± 2.75 μg/mL was detected after the nanocarrier was applied. There were no significant differences (*p* > 0.05) between the two formulations, indicating that the experimental nanocarrier did not cause serious systemic side effects.

**Figure 3:**
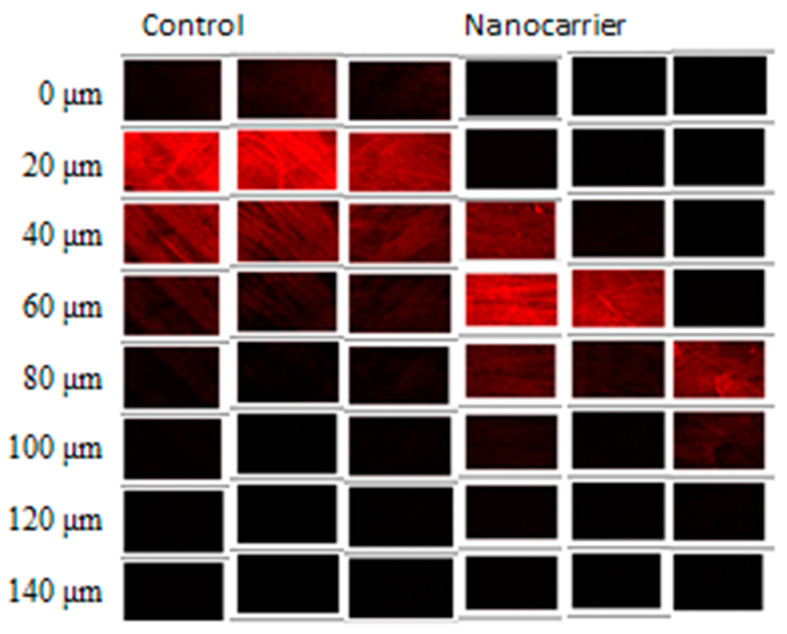
CLSM images of the drug–loaded nanocarrier formulation and control group fluorolabelled with rhodamine B. (sectioned from 0 to 140 μm with 20 μm increments) n = 3.

The CLSM technique is a useful imaging tool for a better understanding of the nanocarriers in delivering therapeutic compounds into tissue layers [23,24]. To evaluate the efficacy of experimental nanocarrier, the CLSM was used to analyze the rhodamine B distribution in the bladder (including depth and intensity) after 1 h application in both nanocarrier and control groups. As shown in Figure 3, the fluorescence of the control group was visualized up to 80 μm, while the nanocarrier was assessed up to 120 μm with a high fluorescence intensity distribution, indicating the penetration potential of nanocarrier formulation. The result was consistent with the previous result of the accumulation of gemcitabine in the bladder tissue increased by about 1.7 times. Furthermore, disease model experiments will be carried out to prove its effectiveness in the future.

## 3. Materials and Methods

### 3.1. Materials and Animals

Gemcitabine hydrochloride was purchased from Scinopharm (Taiwan). Carbitol was obtained from Fluka (Forestparkway, GA, USA). Caproyl 90 (Propylene glycol monocaprylate) and 1,5-pentanediol were acquired from Alfa Aesar (Ward Hill, MA, USA). Acetaminophen, urethane (carbamic acid ethyl ester), methylcellulose and rhodamine B were acquired from Sigma-Aldrich (St. Louis, MO, USA), while benzalkonium chloride and perchloric acid were obtained from Merck Chemicals (Germany). Tetrahydrouridine was purchased from Calbiochem (San Diego, CA, USA), and pentane-sulfonic acid was obtained from Wako (Osaka, Japan). All other solvents and chemicals were of analytical reagent grade.

Animals: Healthy female Sprague–Dawley (SD) rats weighing 200–250 g obtained from BioLASCO Co. Ltd., Taiwan were used for in vitro permeation and in vivo intravesical studies. Animals were housed in plastic cages under standard conditions with a 12 h day-night cycle and standard rodent diet and water can be obtained at will. All animal procedures were performed in accordance with protocols approved by the Kaohsiung Medical University Institutional Animal Care and Use Committee (approved no. 104144)

### 3.2. Drug-Loaded Nanocarrier Preparations

Surfactant and cosurfactant are important components of microemulsions; they will affect the formation, physicochemical properties, and effectiveness of the design formulation. In this study, the appropriate component proportion of cosurfactant of 1,5-pentanediol (X_1_, 10–20%) and carbitol (X_2_, 10–20%), as well as surfactant of benzalkonium chloride (X_3_, 2~8%) in formulation, were investigated by response surface methodology (RSM) with a constrained mixture design. The compositions of model gemcitabine-loaded microemulsion formulations were randomly arranged by Design Experts software (state-Ease Inc, Minneapolis, MN, USA) and are presented in Table 2.

Caproyl 90, surfactant and cosurfactant were mixed in the chosen concentrations, and then water was added portion-wise with continuous vortex for 2 min at room temperature to yield a homogeneous colloid. Then, methylcellulose of 2% was incorporated into the homogeneous colloid for overnight shaking to obtain a well-mixed viscous colloid solution. Then, 1% gemcitabine and/or 0.05% rhodamine B was incorporated into the prepared formulation.

### 3.3. Characterize of Formulations

The cone-plate of viscometer (Model LVDV-II, AMETEK Brookfield, (Middleboro, MA, USA) was used to measure the viscosities of gemcitabine-loaded formulations at a rotation rate of 120 rpm and 37 °C. The particle size analyzer (Malvern Zetasizer 3000HSA)(Malvern Instruments, Ltd., Malvern, UK) with scan angle of 90°, wavelength of 658 nm, and temperature of 25 °C was used to measure the droplet size and polydispersity index of gemcitabine-loaded formulations. Each sample was measured in triplicate and the average and standard deviation were given.

### 3.4. In Vitro Permeation

A previous study has shown that transdermal absorption and local accumulation study through skin can be used to evaluate the permeability and effect of intravesical preparations [25]. Therefore, the modified Franz diffusion cell was used to conduct the permeation study. The effective applied area was 3.46 cm^2^. One milliliter tested formulations equaling 10 mg gemcitabine were applied evenly on the skin surface. The receiver compartment was filled with receptor fluid of pH 7.4 citric acid-sodium phosphate buffer, maintained at 37 °C ± 0.5 °C and stirred at 600 rpm. The bubbles in the receiver compartment had to be removed completely. At predetermined times of 0.5, 1, 2, 3, 4, 6 and 8 h, 1 mL of receptor fluid was withdrawn, and then the same volume of fresh receptor fluid was replaced into the receiver compartment. The transdermal amounts of gemcitabine through the biological membrane at different times were determined by HPLC.

At the end of the experiment, the residual formulation was removed from the donor compartment. The surface of the applied skin was thoroughly washed with distilled water three times to remove any excess formulation and allowed to dry at ambient temperature. The skin was cut into fine pieces; 4 mL of the receptor buffer was added to the skin pieces and homogenized using a Branson homogenizer (Fisher Scientific, Waltham, MA, USA). The homogenized residues were centrifuged at 3000× *g* for 30 min, and then the supernatant obtained after centrifugation was collected and quantified using HPLC.

### 3.5. In Vivo Intravesical Administration of Gemcitabine-Loaded Formulation

Animals were anesthetized by intraperitoneal injection of 1 mL of 50% urethane. The residual urine was evacuated by a slight pressing of the lower abdomen. A PE 50 catheter was carefully inserted into the bladder cavity through the urethra, then, 1.0 mL normal saline was perfused twice to clean the bladder cavity. A 1.0 mL quantity of gemcitabine-loaded experimental formulation or saline solution (as a control group) containing 0.05% rhodamine B was instilled into the bladder cavity and the urethra orifice was ligated using a cotton thread for preventing leaking of the formulation. One hour later, the animals were euthanized, and blood and bladder samples were taken. The blood sample was transferred into heparinized tubes containing 16 μL tetrahydrouridine of 10 mg/mL to prevent degradation of gemcitabine, then centrifuged at 3000× *g* at 4 °C for 10 min. A 200 μL plasma sample was transferred into another tube containing 50 μL internal standard of acetaminophen and 40 μL of 1 M perchloric acid, this mixture was then incubated in an ice bath for 10 min and centrifuged at 16,000× *g* for 10 min, and the resulting supernatant of 20 μL was analyzed.

The drug accumulation amount in the bladder tissue was also measured. The excised tissue was transferred into a tube containing 2 mL normal saline, then the bladder tissue was homogenized at 11,000 rpm for 1 min in an ice bath. Two-milliliter fresh normal saline was used to recover the remaining samples on the homogenizer probe, and then this combined saline fractions solution was then centrifuged at 3000× *g* for 10 min, and the gemcitabine content in the resulting supernatant was analyzed by a modified HPLC from a previous study [25].

### 3.6. Chromatographic Condition

An HPLC was equipped with a Hitachi model L-7100 pump, Hitachi model L-2200 autosampler and a Hitachi model L-4000H detector, and a 250 × 4 mm i.d., 5 μm Lichrocart^®^ C18 column (Merck, German) was used for gemcitabine analysis. The mobile phase composed of acetonitrile and 3 mM pentane-sulfonic acid and 50 mM sodium phosphate aqueous solution (phosphoric acid adjusted the pH to 3.0) and at a ratio of 5 and 95. The flow rate was set at 1.0 mL/min; acetaminophen solution was used as internal standard, and samples were detected at 280 nm. For in vitro samples, the gemcitabine concentration ranged from 1 to 100 1–100 μg/mL with linearity (r^2^ = 0.9994). For plasma samples, the gemcitabine concentration ranged from 0.2 μg/mL to 20 μg/mL with linearity (r^2^ = 0.9993). The limit of quantitation was 0.1 μg/mL. For bladder tissue samples, concentration of gemcitabine ranged from 1 μg g/mL to 100 μg g/mL with linearity (r^2^ = 0.9998). The limit of quantitation was 0.5 μg/mL.

### 3.7. Penetration Depth Measurement

Computerized Zeiss Confocal laser scanning microscopy (CLSM) apparatus (FV 500, Olympus, Tokyo, Japan) was used to acquire the fluorescent images [23,24]. The bladder tissue was collected following in vivo intravesical experiments. The samples were put on the microscope slide for CLSM scanned with an excited wavelength set at 500 nm. X–Z sectioning was used to verify the penetration depth.

### 3.8. Data Analysis

The permeation parameters of transdermal amount, and drug accumulation amounts in tissue at the end of the experiment were used to validate the penetration enhancement effect of experimental formulations. The differences between the gemcitabine-loaded microemulsions were evaluated by ANOVA and Tukey multiple comparison tests using SPSS-Statistic software 22.0 (IBM Corp, Armonk, NY, USA). An average difference of *p*-value < 0.05 is considered significant. The relationships between the independent variables and dependent variables were inspired using RSM provided by Design Expert^®^, a statistical software package version 9.0 (Stat-Ease, Inc., Minneapolis, MN, USA). The optimal mixture design of the experiment method was to fit the empirical model, test the sufficiency of the fitted equation, image the figure of the response surface, and decide the optimal range of the component ratio.

## 4. Conclusions

The purpose of this study was to develop nanocarriers for chemotherapeutic agents of intravesical administration. The in vitro permeation study and in vivo intravesical instillation study were conducted to prove the efficacy of nanocarriers. In the in vitro permeation study, the transdermal and accumulation amounts in the tissue of the nanocarrier formulation remarkably enhanced 14.8~33.0-fold and 1.53~14.1-fold, respectively. For in vivo intravesical insfusion, when using nanocarrier as a delivery carrier, the drug accumulation amount in bladder tissue was remarkably enhanced. The penetration depth also increased from 60 μm up to 120 μm; moreover, there was no significant increase in drug plasma concentration. The result highlights that the nanocarrier formulation could be considered as a promising drug delivery system for the intravesical administration of gemcitabine.

## Figures and Tables

**Table 1:** The composition and physicochemical properties of model gemcitabine-loaded formulations provided mixture design.

	X_1_ Code	X_2_ Code	X_3_ Code	Size nm			PDI			Viscosity (cps)		
F01	0.50	0.43	0.07	57.4	±	0.1	0.05	±	0.00	481.0	±	6.5
F02	0.50	0.43	0.07	55.3	±	5.3	0.19	±	0.01	483.0	±	11.0
F03	0.88	0.00	0.12	163.5	±	2.4	0.35	±	0.02	407.2	±	15.7
F04	0.95	0.04	0.01	10.5	±	1.4	0.20	±	0.03	661.1	±	13.8
F05	0.40	0.60	0.00	76.2	±	3.1	0.20	±	0.01	464.7	±	5.3
F06	0.24	0.76	0.00	141.9	±	14.1	0.11	±	0.01	326.6	±	14.9
F07	0.23	0.63	0.14	168.0	±	46.1	0.14	±	0.01	330.9	±	20.3
F08	0.00	0.92	0.08	165.9	±	40.5	0.13	±	0.02	274.1	±	8.8

X_1_: 1,5-pentanediol; X_2_: carbitol; X_3_: benzalkonium chloride; PDI: polydispersity index.

**Table 2:** Variables and intervals selected to perform the constrained mixture design.

Variables	Code Level Low	Code Level High
X_1_: 1,5-Pentanediol 10~20%	0.0	0.6
X_2_: Carbitol 10~20%	0.0	1.0
X_3_: Benzalkonium chloride 1.5~3%	0.0	0.6

The amounts of gemcitabine, capryol 90 and methylcellulose in formulations were fixed at 1%, 5% and 2% respectively. The total amount of three variables of X_1_, X_2_ and X_3_ in formulation was 32%.
